# The Neanderthal Meal: A New Perspective Using Faecal Biomarkers

**DOI:** 10.1371/journal.pone.0101045

**Published:** 2014-06-25

**Authors:** Ainara Sistiaga, Carolina Mallol, Bertila Galván, Roger Everett Summons

**Affiliations:** 1 Department of Geography and History, University of La Laguna, Tenerife, Spain; 2 Department of Earth, Atmospheric and Planetary Sciences, Massachusetts Institute of Technology, Cambridge, Massachusetts, United States of America; 3 Instituto Universitario de Bio-Orgánica Antonio González, La Laguna, Tenerife, Spain; ICREA at the Universitat Autònoma de Barcelona, Spain

## Abstract

Neanderthal dietary reconstructions have, to date, been based on indirect evidence and may underestimate the significance of plants as a food source. While zooarchaeological and stable isotope data have conveyed an image of Neanderthals as largely carnivorous, studies on dental calculus and scattered palaeobotanical evidence suggest some degree of contribution of plants to their diet. However, both views remain plausible and there is no categorical indication of an omnivorous diet. Here we present direct evidence of Neanderthal diet using faecal biomarkers, a valuable analytical tool for identifying dietary provenance. Our gas chromatography-mass spectrometry results from El Salt (Spain), a Middle Palaeolithic site dating to ca. 50,000 yr. BP, represents the oldest positive identification of human faecal matter. We show that Neanderthals, like anatomically modern humans, have a high rate of conversion of cholesterol to coprostanol related to the presence of required bacteria in their guts. Analysis of five sediment samples from different occupation floors suggests that Neanderthals predominantly consumed meat, as indicated by high coprostanol proportions, but also had significant plant intake, as shown by the presence of 5β-stigmastanol. This study highlights the applicability of the biomarker approach in Pleistocene contexts as a provider of direct palaeodietary information and supports the opportunity for further research into cholesterol metabolism throughout human evolution.

## Introduction

Dietary differences between Neanderthals (extinct human species that lived in Eurasia between ca. 230.000 to 40.000 years ago) and anatomically modern humans have been claimed to be one of the possible causes of their disappearance [1]. The complex spectrum of food sources exploited by the latter could have represented an adaptive advantage compared to a more restricted Neanderthal diet based on high meat intake.

Major progress has been made in Neanderthal dietary reconstructions by the combination of stable carbon and nitrogen isotopes [2]–[4]. However, since these data only reflect the principal sources of protein intake, the role of plants is underestimated and Neanderthals continue to be pictured as top-level carnivores. Supporting information comes from the high occurrence of faunal remains in Neanderthal sites showing that they were expert hunters of large herbivores with short prey variability [5].

New evidence challenging this view comes from dental calculus analysis and microfossils trapped in Neanderthal teeth [6]–[8]. These data present a completely different image of Neanderthals as a population that exploited and cooked a wide range of plant species. Further evidence comes from palaeobotanical remains found in archaeological deposits such as Kebara Cave or Amud Cave [9], [10]. However, the preservation potential of plant remains is high in the Middle East, but low in the rest of the land inhabited by Neanderthals. In sum, except for the evidence of entrapped microfossils and organic residues in Neanderthal teeth, all previous palaeodietary reconstructions have been based on indirect evidence where preferential or selective preservation plays a key role.

We have obtained the first direct evidence of animal and plant intake by Neanderthals based on identification of human faecal biomarkers in archaeological sediments. This method has proved be an increasingly valuable tool in the identification of the likely source of faecal matter and has successfully been applied in more recent archaeological contexts [11]–[16]. It provides direct evidence of the digestive physiology and diet of the source organisms and provides critical data to assess the origin of faecal deposits [11], [12].

We focus on sterols and stanols; lipids that are known to be relatively stable through food chains and during early diagenetic processes [17], [18]. More specifically, 5β-stanols can be used as faecal biomarkers because they are uniquely formed in the intestinal tract of most higher mammals during metabolic reduction of cholesterol and phytosterols. Moreover, their relative proportions are indicative of dietary preferences [11], [19]. The main actors of this microbial conversion still have to be elucidated [20], but a few cholesterol-reducing strains have been isolated [21]–[24] and only one from human faeces [24]. Genes or enzymes involved in this metabolism are still unknown [25].

GC-MS analyses were carried out using a sensitive and selective detection method, that is, multiple reaction monitoring (MRM). We used precursor-product mass spectrometric transitions for sterol and stanols in combination with gas chromatographic retention times to establish the composition of steroidal lipids isolated from combustion structures from El Salt Unit X (Appendix S1).

## Materials and Methods

**Figure 1:**
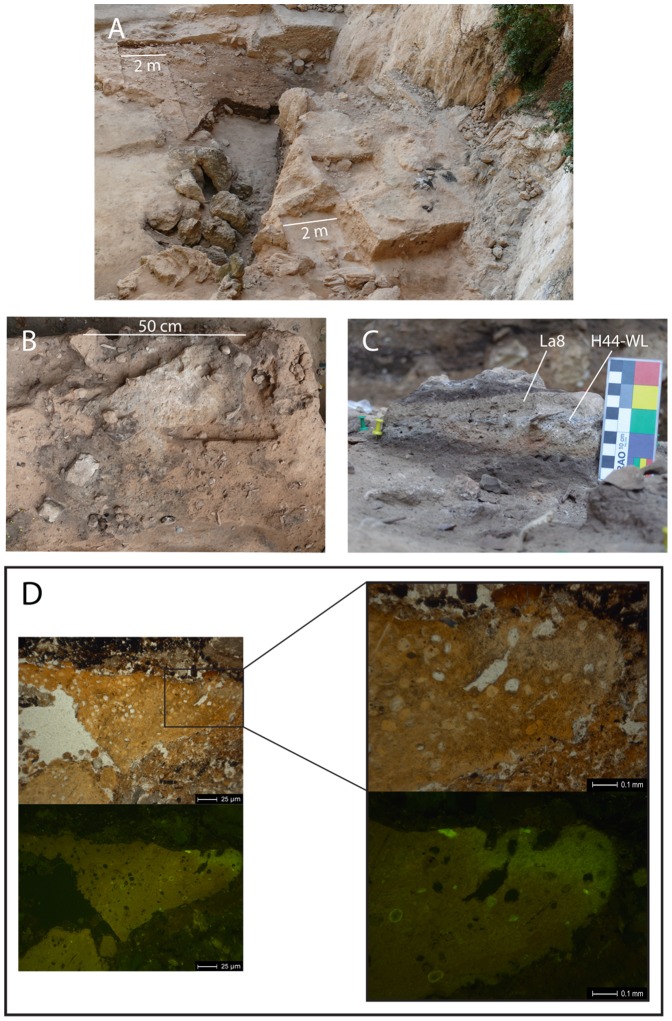
Archaeological and micromorphological context. A) El Salt site during excavation; B) Field photograph showing a detail of exposed combustion structure H44 (white sediment corresponds to the top ash layer). The black sediment to the left belongs to an overlying combustion structure (H32). C) Field photograph of sediment block showing the facies described in the text in microstratigraphic succession. D) Microphotographs of a slightly burned coprolite of putative human origin identified in El Salt Stratigraphic Unit X (sample SALT-08-13). The images under plane polarized light show the pale brown color and massive structure of the coprolite, as well as the common presence of inclusions, which are possibly parasitic nematode eggs or spores. Views under blue light fluorescence (black background) shows autofluorescence indicative of high phosphate content.

The archaeological setting of El Salt (Alicante, Spain), is a Middle Palaeolithic open-air site (Fig. 1A) that has yielded evidence of recurrent Neanderthal occupation dated between 60.7±8.9 and 45.2±3.4 Ka and is under current investigation [26], [27].

The sediment samples belong to the stratigraphic unit under current excavation (S.U. X) and are associated with archaeological remains and sedimentary facies were excavated, sampled and documented according to a methodology geared to the identification of human occupation episodes. The selected combustion features belong to diachronous archaeological facies associations.

Our evidence for animal and plant intake comes from the top of the white layer of combustion structure H44 and its overlying deposit (Fig. 1C). This ash layer has shown to be microstructurally well preserved, not reworked by bioturbation, and is overlain by a sandy clayey sediment (La8) with a sharp contact. Several millimetric phosphatic coprolites with micromorphological features resembling those reported for human coprolites [28] were identified in thin sections manufactured from oriented blocks of sediment collected from stratigraphic unit X, near the samples reported here. These coprolites are autofluorescent under blue light, have a massive groundmass and contain numerous inclusions, possibly the remains of parasitic nematode eggs or spores [29], [30]. (Fig. 1D).

Samples were collected by hand during surface excavation of El Salt Neanderthal site. About 10 g of sediment were scooped out using metal tools rinsed in methanol (MeOH) and dichloromethane (DCM), wrapped in aluminium foil, inserted in plastic bags and stored in a freeze room. In the laboratory, a half of the samples were pulverized to a fine powder using an agate mortar and pestle. All laboratory equipment was rinsed with high-purity acetone, MeOH and DCM in between samples to avoid cross contamination. We also analyzed fresh primate stool as control samples for biomarker identification. All glassware, aluminium foil, silica, quartz wool and quartz sand were combusted at 500°C for at least 12 hours to remove organic contamination, whereas metal tools were rinsed in MeOH and DCM. About 5 g of homogenized sediment samples were freeze dried and ultrasonically extracted 4 times with DCM/MeOH (3∶1 v/v) at 40°C for 20 minutes to obtain a total lipid extract (TLE).

Solvent was removed from the TLEs under a constant low-velocity stream of N_2_ gas using a TurboVAP and dried samples were stored under nitrogen in glass vials with Teflon-lined caps. The TLE was then derivatised to allow identification of sterols to their trimethyl silyl ethers (TMS) using a mixture of 25 µl pyridine and 25 µl N,O-bis [trimethylsilyl]trifluoroacetamide containing 1% of trimethylchlorosilane solution (BSTFA+ 1% TCMS; SIGMA).

Stenols and stanols were analyzed by GC–MRM–MS (Supplementary methods S1 in Appendix S1), using predetermined precursor–product reactions (Table S1 in Appendix S1). MRM–MS is a sensitive analytical technique performed in a double-focusing magnetic sector mass spectrometer and measures fragmentation reactions taking place in the first field free region of the mass analyser. This approach affords high signal to noise ratio and elevated selectivity of targeted lipid classes, enabling the identification of compounds that are normally unresolved or co-eluting in complex or heavily biodegraded mixtures or in very low abundances [18]. The sterols in the samples we analysed co-occurred with many other compounds in a complex mixture of overlapping peaks. The GC-MRM-MS analysis allowed us to visualise the compounds as a suite of cleanly resolved peaks from which we could reliably determine peak areas at low levels of sample consumption. GC alone would never give the same confidence for identification or quantification. We did not attempt a direct comparison with more conventional GC-MS although it is probable we could have identified their presence GC-MS in Scan or SIM mode if more material had been available to work with. Identification of compounds was achieved by comparison with reference samples and literature.

We have followed a conventional organic geochemistry protocol consistent with our objective. As this study targets faecal sterols, we do not include total sterol content in our results. Work conducted independently of this study has confirmed that our GCMS analyses, when quantitation is required, are reproducible to better than ±5%.

Adscription of the samples to a human origin was carried out based on previously standardized ratios [11], [32], [36]. Therefore, no statistical treatment was necessary.

## Ethics Statement

We declare that no living animals were used in this research. The soil samples used for biomarker and micromorphological analyses were collected from the archaeological site of El Salt, Alicante, Spain. The archaeological team leaded by B. Galván conducted the excavations under a government permit and following the Spanish heritage law (No. 16/1985, 25 june). All excavated material including the sedimentary material is interpreted as archaeological material so no further permits are required for the presented study.

## Results

**Figure 2:**
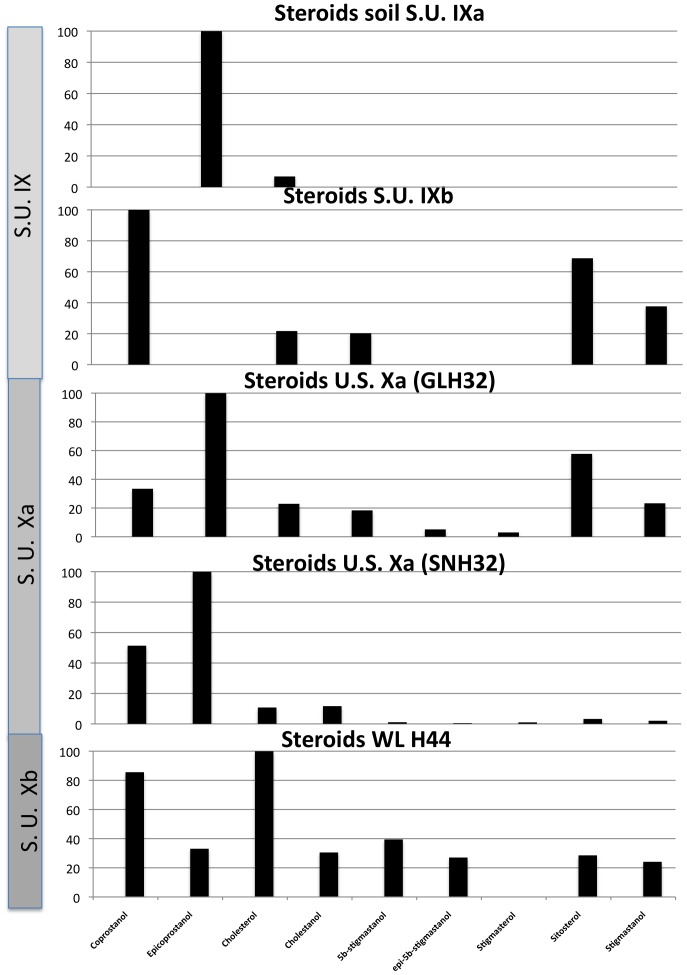
Sterol fraction histograms. Sterol fraction histograms from the samples reported here that yielded faecal matter. Relative concentrations are expressed as % of the largest peak compared to peak areas in the TIC.

**Figure 3:**
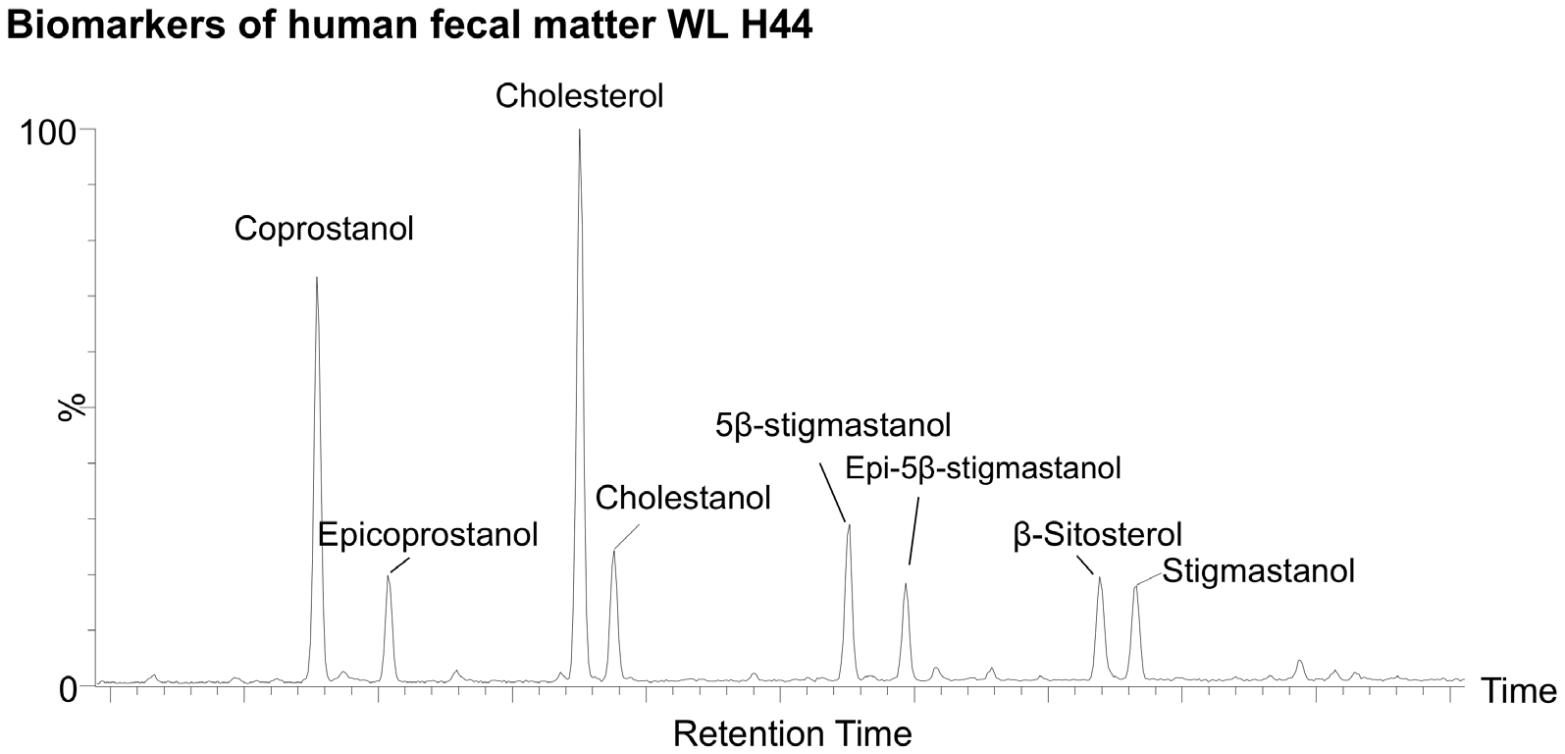
Partial chromatogram showing evidence for plant intake. Partial chromatogram of summed GC-MS transition for C_27_+C_28_+C_29_ sterols and stanols from the oldest sample (WL H44). Retention peaks matched those of reference samples of fresh faecal matter from primates and herbivores.

Our preliminary results from four of five samples show an alcohol fraction clearly dominated by coprostanol and its diagenetic product epicoprostanol, with some presence of stigmasterol, sitosterol and cholesterol (Fig. 2). A fifth sample (WL H44) also yielded higher proportions of cholesterol and coprostanol. However, significant occurrence of phytosterol metabolites, namely 5β-stigmastanol and 5β-epistigmastanol, unambiguously record the ingestion of plants (Fig. 3). Hydrocarbon analyses of the same sample did not show alkanes with odd over even predominance, diagnostic for the presence of plant leaf waxes, thereby excluding the surrounding vegetation as a direct source of these compounds.

Cholesterol and phytosterols were detected in control soils sampled within a distance of >1 m from the combustion structure. However, we did not identify the presence of any 5β-stanols in these control samples.

Because cholesterol to coprostanol conversion is not unique to human beings [31]–[35] several ratios originally used as indicators of sewage pollution have been proposed to identify the likely source of faecal matter [11], [36]. Here we used the ratio proposed by Bull and coworkers [11] [coprostanol+epicoprostanol/5β-stigmastanol+epi-5β-stigmastanol] in order to distinguish between omnivore, pig/human (>1), and herbivore species (<1). The value obtained using this ratio (1.78) suggests a human origin.

## Discussion and Conclusions

Even though some studies [13], [37]–[41] have reported small concentrations of coprostanol in soils generated by microbial action in reducing conditions, this represents a minor pathway in cholesterol environmental conversion, while the vast majority of cholesterol will be transformed into cholestanol. In any case, our samples from the same level and vertical sampling test did not show a regular presence of 5β-stanols or cholestanol, indicating that the presence of faecal biomarkers in our samples is not due to environmental contamination.

Coprostanol is formed through microbial hydrogenation of its precursor Δ^5^-sterol cholesterol by the specific bacteria present in the intestinal tract of higher mammals. It is formed from both ingested and *de novo* biosynthesized cholesterol [11], [41]. The other stanols represent direct dietary input of plant sterols, since humans cannot synthesize them. The sterol composition of an animal's diet, and the amount of plant material in particular, has a clear impact on stanol profile. Analyses reported for other animals such as dogs and birds contain only trace amounts of 5β-stanols probably due to the lack of sterol-reducing bacteria [31]–[35].

In humans, the conversion of cholesterol to coprostanol is bimodal with a vast majority of high converters and a minority of low to inefficient converters (coprostanol content representing less than one-third of faecal sterol content) [42], [43]. The efficiency of cholesterol conversion depends mainly from the abundance of cholesterol-reducing bacteria [25]. This microbial action starts in humans during the second half of the first year of life [44] and would facilitate the elimination of excess cholesterol from the body, decreasing the risk of cardiovascular disease [45]–[46].

The predominance of coprostanol and a near absence of 5β-stigmastanol among our samples indicate that the Neanderthals from El Salt had a meat-dominated diet. However, this must be viewed from the perspective that animal tissues contain far higher abundances of the precursor sterols than do plant tissues. Normally, the sterol content of meat is 3 times higher, gram for gram, of fresh plants such as cabbage [47], [48]. Nevertheless, higher plant intake would be indicated by the presence of 5β-stigmastanol. For example, consumption of tubers would yield a significant proportion of this plant's digestion products. This disagrees with claims of preservation bias hampering our ability to identify consumption of tubers (underground storage organs) and other plants [6]. In fact, the tandem occurrence of 5β-stigmastanol from the oldest sample (WL H44) points to some plant intake (Fig. 3). According to Buck and Stringer [49], this could either represent direct consumption of plants or stomach contents of animals that had consumed plants.

Taken together, these data suggest that the Neanderthals from El Salt consumed both meat and vegetables, in agreement with recent hypotheses based on indirect evidence. Future studies in Middle Palaeolithic sites using the faecal biomarker approach will help clarify the nature, role and proportion of the plant component in the Neanderthal diet, and allow us to assess whether our results reflect occasional consumption or can be representative of their staple diet. Also, this data represents the oldest positive identification of human faecal matter, in a molecular level, using organic geochemical methods.

Besides having corroborated our method and obtained the first evidence of an omnivorous Neanderthal diet from faeces, our results also have implications regarding digestive systems and gut microbiota evolution. Approaching the evolution of the human digestive system is difficult because there is no fossil record indicating soft tissue preservation. Our results show that Neanderthals, like anatomically modern humans, have a high rate of conversion of cholesterol to coprostanol due to the presence of bacteria capable of doing so in their guts. Further research will allow us explore this issue in the context of human evolution.

## Supporting Information

Appendix S1 Supplementary methods S1, *Gas Chromatography –Multiple Reaction Monitoring – Mass Spectrometry (GC–MRM–MS).* Table S1, MRM-GC-MS Precursor-Product Transitions.
(DOCX)
