# Supplementary material for: The Neanderthal Meal: A New Perspective Using Faecal Biomarkers
Source: PLoS One. 2014 Jun 25;9(6):e101045. doi: 10.1371/journal.pone.0101045 (PMC4071062; doi:10.1371/journal.pone.0101045)
Supplement: Appendix S1 — Supplementary methods S1, Gas Chromatography –Multiple Reaction Monitoring – Mass Spectrometry (GC–MRM–MS). Table S1, MRM-GC-MS Precursor-Product Transitions. (DOCX) [file pone.0101045.s001.docx]

Appendix S1. Supplementary Materials:

Supplementary Methods S1

Table S1

**Supplementary methods S1.**

*Gas Chromatography –Multiple Reaction Monitoring – Mass Spectrometry (GC–MRM–MS)*

Sterols were analyzed in full scan and MRM modes using a Micromass Autospec Ultima mass spectrometer interfaced to an Agilent 6890 N gas chromatograph. The GC was fitted with a DB-1 fused silica capillary column (60 m; 0.25 mm I.D.; 0.25 μm film thickness; J&W Scientific) and He was used as the carrier gas. Samples, dissolved in the Py+BSTFA reagents, were introduced to the GC by a PTV injector in splitless mode (ramped from 60ºC to 330ºC with 720º/minute upon injection). The GC temperature program was: 60°C (2min) to 150°C at 10°C min^-1^, to 315°C (held 24 min) at 3°C min^-1^. The Autospec source was operated in electron ionization (EI, 70 eV) mode at 250° C, with 8 kV accelerating voltage for MRM. Full scan analyses were conducted over a range of m/z 50–600. Data were acquired and processed using MassLynx 4.0 (Micromass Ltd).

**Table S1**. MRM-GC-MS Precursor-Product Transitions

| **Common Name** | **IUPAC name** | **Molecular Mass TMS Derivative** | **Parent** | **Product** |
| --- | --- | --- | --- | --- |
| Coprostanol | 5β-cholestan-3β-ol | 460 | 370.30 | 215.20 |
| Cholesterol | (3β) cholest-5-en-3β-ol | 458 | 368.30 | 213.30 |
| Cholestanol | 5α-cholestan-3β-ol | 460 | 370.30 | 215.20 |
| Ergosterol | Ergosta-5,7,22-trien-3β-ol | 472 | 382.30 | 213.30 |
| Ergostanol | (1S,2S,10R,11S,14R,15R)-14-((2R,5R)-5,6-dimethylheptan-2-yl)-2,15-dimethyltetracyclo(8.7.0.0^{2,7}.0^{11,15})heptadecan-5-ol | 474 | 384.30 | 215.20 |
| 5β-stigmastanol | 24-ethyl-5β-cholestan-3β-ol | 488 | 398.30 | 215.20 |
| Stigmasterol | (3*S*,8*S*,9*S*,10*R*,13*R*,14*S*,17*R*)-17-((*E*,2*R*,5*S*)-5-ethyl-6-methylhept-3-en-2-yl)-10,13-dimethyl-2,3,4,7,8,9,11,12,14,15,16,17-dodecahydro-1*H*-cyclopenta(*a*)phenanthren-3-ol | 486 | 396.30 | 213.20 |
| Β-Sitosterol | 24-ethylcholestan-3β-ol | 486 | 396.30 | 213.20 |
